# Management of follicular thyroid carcinoma

**DOI:** 10.1530/ETJ-24-0146

**Published:** 2024-10-16

**Authors:** Haruhiko Yamazaki, Kiminori Sugino, Ryohei Katoh, Kenichi Matsuzu, Wataru Kitagawa, Mitsuji Nagahama, Aya Saito, Koichi Ito

**Affiliations:** 1 Department of Breast and Thyroid Surgery, Yokohama City University Medical Center, Yokohama City, Kanagawa, Japan; 2 Department of Surgery, Ito Hospital, Shibuya-ku, Tokyo, Japan; 3 Department of Pathology, Ito Hospital, Shibuya-ku, Tokyo, Japan; 4 Department of Surgery, Yokohama City University School of Medicine, Yokohama City, Kanagawa, Japan

**Keywords:** differentiated thyroid cancer, thyroid cancer, thyroidectomy

## Abstract

Follicular thyroid carcinoma (FTC) is the second most common histological type of thyroid carcinoma. This review aims to summarize the available evidence and guidelines and provide an updated consensus regarding the management of FTC. The cytoarchitectural features of FTC are similar to those of follicular adenoma (FA), and it is difficult to preoperatively distinguish between FA and FTC. For nodules with Bethesda class III–V cytology, molecular test results (if available) should be considered before the operation. However, it should be noted that molecular tests are not available in all countries. The goals of initial surgical therapy for patients with FTC are to improve overall and disease-specific survival, reduce the risk of persistent/recurrent disease and associated morbidity, and permit accurate disease staging and risk stratification while minimizing treatment-related morbidity and unnecessary therapy. Previous studies have reported some prognostic factors such as distant metastasis, age, tumor size, vascular invasion, *TERT* promoter mutation, and histological subtype. In particular, the degree of vascular invasion is becoming increasingly important. Evaluating these prognostic factors is essential for prognostic prediction and precise management of patients with FTC. Recurrence and distant metastasis of FTC are treated with radioactive iodine (RAI). However, some FTCs become refractory to RAI. Multi-tyrosine kinase inhibitors such as sorafenib and lenvatinib are utilized for treating RAI-refractory FTCs. In addition, given that renin–angiotensin system (*RAS*) is the most common driver gene for FTC, it is also important to develop RAS inhibitors.

## Introduction

Thyroid carcinoma is the most common endocrine-related malignancy, with an incidence of 821,214 new cases diagnosed in 2022 (World Health Organization. Cancer Today–GLOBOCAN2020. Available from: https://gco.iarc.fr/today/home. (Accessed 18 March 2024)). Follicular thyroid carcinoma (FTC) is the second most common histological type of thyroid carcinoma (1). The third edition of the World Health Organization (WHO) classification divides FTCs into two major categories based on their degree of invasiveness. Accordingly, minimally invasive FTCs demonstrate limited capsular and/or vascular invasion that is not grossly visible and can be identified only under a microscope, whereas widely invasive FTCs demonstrate widespread infiltration into adjacent thyroid tissues and/or blood vessels (2). Subsequently, the fourth edition of the WHO classification divides minimally invasive FTCs into two subgroups: minimally invasive FTC (capsular invasion only) and encapsulated angioinvasive FTC (3). The capsule of a widely invasive FTC may be entirely obliterated or focally intact. Widely invasive FTC shows extensive invasion of the thyroid and often extra-thyroidal soft tissues. Vascular invasion is often prominent in widely invasive FTC. However, the classification of widely invasive FTC is not determined by the presence or absence of vascular invasion (3).

Most patients with FTC first undergo hemithyroidectomy with a diagnosis of follicular or adenomatous nodules. Since the prognosis of patients with widely invasive FTC is significantly poorer compared to patients with minimally invasive FTC, completion total thyroidectomy with radioactive iodine (RAI) therapy for widely invasive FTC to detect the appearance of distant and local metastasis is strongly recommended in clinical practice guidelines (4, 5). However, it is not uniformly recommended for minimally invasive FTC without distant metastasis. The goals of initial surgical therapy for patients with FTC are to improve overall and disease-specific survival, reduce the risk of persistent/recurrent disease and associated morbidity, and permit accurate disease staging and risk stratification while minimizing treatment-related morbidity and unnecessary therapy (4).

Follicular thyroid cells produce thyroglobulin (Tg), which can be used as a marker. Serum Tg levels can be elevated in most thyroid diseases, but it is not a sensitive or specific test for thyroid cancer. Therefore, routine measurement of serum Tg for the initial evaluation of thyroid nodules is not recommended (4). However, it may be useful for estimating the presence or absence of distant metastasis (6). After the initial treatment, measurement of serum Tg levels is an important modality for monitoring patients with residual or recurrent disease (4). In addition, an increase or reappearance of Tg antibody (TgAb) during follow-up is highly suggestive of recurrence or persistence (7).

Recurrence and distant metastasis of FTC are treated with RAI. However, some FTCs become refractory to RAI. Multi-tyrosine kinase inhibitors such as sorafenib and lenvatinib are utilized for treating RAI-refractory FTCs (8, 9).

Numerous guidelines regarding differentiated thyroid carcinoma have been formulated by professional bodies (4, 10, 11, 12). However, almost all evidence is based on studies of papillary thyroid carcinoma (PTC). Unlike PTC, the relatively low incidence and limited number of large-scale studies of FTC have resulted in a paucity of high-quality evidence to reach a consensus on management. This review aims to summarize the available evidence and guidelines and provide an updated consensus regarding the management of FTC.

## Etiology

Iodine deficiency is considered a risk factor based on studies demonstrating a higher incidence of FTC and an increased FTC-to-PTC ratio in iodine-deficient areas relative to iodine-sufficient areas and series, demonstrating a decrease in FTC incidence with iodine supplementation (13, 14). FTC can occur in the setting of syndromes predominantly defined by extrathyroidal manifestations, including *PTEN* hamartoma tumor syndrome, DICER1 syndrome, Werner syndrome, and Carney complex (15, 16).

## Pathogenesis

The most common somatic mutations in FTC are *RAS* point mutations, which are reported in up to half of FTCs, with an overall rate of approximately 30% in published studies (17). *NRAS* mutations are the most common, followed by *HRAS* and *KRAS* mutations (17). Most *NRAS* and *HRAS* mutations involve codon 61, whereas most *KRAS* mutations involve codons 12/13 (18). *PAX8::PPARG* rearrangements occur in 10–40% of FTCs, with the frequency showing more geographic variability than *RAS* mutations (17, 19). *RAS* mutations and *PAX8::PPARG* rearrangements are mutually exclusive. In contrast to *RAS*-mutant FTC, FTC with *PAX8::PPARG* rearrangements have been reported to occur in younger patients and are smaller in size but with more overt invasion (20, 21).

## Diagnosis

The cytoarchitectural features of FTC are similar to those of follicular adenoma (FA). Therefore, FTC may be suspected but generally cannot be diagnosed using a fine needle aspiration biopsy (FNAB). Fundamentally, it is difficult to preoperatively distinguish between FA and FTC. Distinguishing between an FTC (malignant tumor with capsular and/or vascular invasion) and a benign FA (no invasion) requires pathological examination of the tumor capsule after tumor excision. The risk of malignancy (ROM) with an FNAB reading of follicular neoplasm is 23–34% (Bethesda IV) and 13–30% with AUS/FLUS (Bethesda III) (22). Molecular testing is described for the usual management in the Bethesda System for Reporting Thyroid Cytopathology. However, some FAs have pathogenic mutations, including *RAS* and *TERT* promoter mutations (23). Hirokawa *et al.* investigated the ROM based on the following four findings: cytological findings favoring malignancy, ultrasound (US) findings of high suspicion, tumor size ≥30 mm, and tumor volume-doubling rate ≥1.0/year (24). Follicular neoplasms without any of these four findings showed a ROM of only 1.0% (24).

## Initial treatment strategy

According to the American Thyroid Association (ATA) guidelines, surgery may be considered for growing nodules that are benign after repeat FNA if they are large (>4 cm), cause compressive or structural symptoms, or are based upon clinical concern (4). Similarly, surgery may be appropriate in the following scenarios: symptomatic nodular thyroid disease, as an alternative to the minimally invasive technique (MIT) and RAI therapy; nodules that have been classified as benign on cytology and/or low risk on US and become symptomatic over time; and nodules with indeterminate cytology (Bethesda classes III and IV) that are not suitable for active surveillance (i.e. large size, high suspicion of malignancy on US, symptomatology) in the European Thyroid Association guideline (25). MITs are outpatient procedures performed under US guidance for the non-surgical management of thyroid lesions, which include ethanol ablation and thermal ablation using various energy sources (e.g. laser, radiofrequency, microwaves, or high-intensity focused ultrasound) (25). For nodules with Bethesda classes III–V cytology, surgery allows for a definitive diagnosis (26). Molecular test results (if available) should be considered before the operation (22). In fact, a randomized trial performed by Livhits *et al.* revealed that molecular tests displayed high specificity for Bethesda classes III–V nodules and allowed 49% of patients with these nodules to avoid diagnostic surgery (27). However, it should be noted that molecular tests are not available in all countries. In contrast, total thyroidectomy is performed in patients with a prior diagnosis of distant metastasis or bilateral thyroid tumors suitable for surgery.

## Clinical prognostic factors

### Distant metastasis

Distant metastasis is a negative prognostic factor for FTCs (28, 29, 30, 31). As with PTC, distant metastases are primarily found in the lungs and bones, but metastases to the liver, brain, and other sites also occur (32). Even in minimally invasive or encapsulated FTC, 1–9% of patients have distant metastasis at the time of initial treatment (29, 31, 32, 33). In contrast, patients with widely invasive FTC have higher rates of distant metastasis at the time of initial treatment, ranging from 8% to 45% (28, 34, 35, 36). O’Neil *et al.* reported that distant metastases were encountered more frequently in patients with encapsulated angioinvasive and widely invasive FTCs, with the majority being asymptomatic and confirmed at the time of RAI remnant ablation (35). Yamazaki *et al.* reported that 16 (35%) of 46 distant metastases were diagnosed after the initial RAI therapy, which was performed as ablation in 474 patients with FTC (37). In contrast to PTC, locoregional recurrence is rare in FTC (38). Therefore, controlling distant metastasis is important for avoiding death in patients with FTC (39).

### Age

In 2012, Sugino *et al.* investigated the prognostic factors of 251 patients with minimally invasive FTC (29). The study showed that there were significantly more patients with preoperative distant metastases in the group of patients who were ≥45 years of age (*P* = 0.0004). Among the 251 patients with FTC, the cause-specific survival (CSS) rate was significantly poorer in patients ≥45 years of age. In the 229 patients with M0 FTC, the distant metastasis-free survival (DMFS) rate was also significantly lower in patients ≥45 years of age. In addition, age was identified as the only independent significant prognostic factor for poorer CSS (odds ratio = 11.5, 95% CI: 0.0303–0.1390, *P* = 0.0007) and a factor related to DMFS (odds ratio = 14.3, 95% CI: 0.0205–0.0689, *P* = 0.0002) in the M0 group.

In 2021, Yamazaki *et al.* investigated the impact of a change in cutoff age from 45 to 55 years among 478 patients with minimally invasive and encapsulated angioinvasive FTC (30). The study included 192 (40%) patients of <45 years, 75 (16%) patients aged 45–55 years, and 211 (44%) patients aged ≥55 years. The 10-year CSS rates of patients aged <55 and ≥55 years were 100% and 95.5% (*P* = 0.010), respectively. None of the patients aged <55 years died during the follow-up period. Among the 458 patients with M0 FTC, the 10-year disease-free survival (DFS) rates of patients aged <45 years of age, 45–55 years, and ≥55 years of age were 97.0%, 95.5%, and 86.4%, respectively. Patients ≥55 years of age had significantly worse DFS than patients in other age groups (*P* = 0.008). It is uncertain whether a cutoff age of 55 years is appropriate for patients with FTC (30, 40). Further studies are needed to determine the optimal cutoff age specific to FTC.

### Tumor size

Podda *et al.* investigated the differences in clinical relevance between 42 patients with minimally invasive FTC and 29 patients with widely invasive FTC (41). The study indicated that tumor size >4 cm was the only independent predictor of overall recurrence and influenced DFS (odds ratio = 6.750, 95% CI: 1.01–44.92, *P* = 0.048).

Ito *et al.* investigated the prognosis and prognostic factors associated with 292 patients with minimally invasive FTC (42). In a multivariate analysis of 292 patients, a tumor >4 cm was an independent prognostic factor (odds ratio = 25.641, 95% CI: 1.037–50.000, *P* = 0.0474) for CSS. In addition, in a multivariate analysis of 285 patients with M0, a tumor >4 cm was also an independent prognostic factor (odds ratio = 3.509, 95% CI: 1.092–11.364, *P* = 0.0352) for DFS.

In the study mentioned above (30), the 10-year DFS rates of patients with tumor sizes ≤40 mm and >40 mm were 95.8% and 88.9%, respectively (*P* < 0.001). Furthermore, tumor size >40 mm was identified as an independent negative prognostic factor (hazard ratio (HR) = 3.460, 95% CI: 1.460–8.201, *P* = 0.005) for DFS.

Recently, Ginzberg *et al.* revealed that a tumor size of ≥4 cm and ≥1 other high-risk feature (e.g. extrathyroidal extension) together yielded worse survival than either a tumor size of ≥4 cm or other high-risk features alone (43). The study concluded that concomitant high-risk features confer worse survival than a large tumor size alone, and a 4 cm cutoff is not associated with the greatest increase in risk. In another study, widely invasive FTC with massive extrathyroidal extension had a poorer prognosis (34). Regardless, caution should be exercised in regard to FTC-related mortality and recurrence among patients with large tumors. Although it is more difficult to assess margins in FTC with larger tumors, the extent of invasion should also be assessed.

### Vascular invasion

The fourth edition of the WHO classification divides minimally invasive FTCs into the following two subgroups based on O’Neill’s study: capsular invasion only minimally invasive FTCs and encapsulated angioinvasive FTCs (35). As the presence of VI significantly affects the prognosis of FTC, it is appropriate to divide minimally invasive FTCs into two groups according to the presence or absence of VI (34).

Ito *et al.* classified cases with ≥4 vascular invasions as having extensive vascular invasion (42). Among the 292 patients, extensive vascular invasion was an independent prognostic factor (odds ratio = 13.699, 95% CI: 1.085–166.667, *P* = 0.0430) for CSS. In addition, among the 285 M0 patients, extensive vascular invasion was an independent prognostic factor (odds ratio = 5.405, 95% CI: 1.715–16.95, *P* = 0.0040) for DFS.

Most previous studies used four vascular invasions as the value to analyze the correlation between the prognosis of thyroid cancer and vascular invasion (42, 44, 45). Yamazaki *et al.* investigated the association between the extent of VI and the outcome of encapsulated angioinvasive FTC (31). The study included 264 patients with encapsulated angioinvasive FTC and reported that two vascular invasions were the new optimal cutoff value for both CSS and DFS based on a receiver operating characteristic curve analysis. The 10-year CSS rates of patients with one vascular focus and ≥2 vascular invasion foci were 100% and 94.5% (*P* = 0.042), respectively. In addition, the 10-year DFS rates of patients with one vascular focus and ≥2 vascular invasion foci were 94.9% and 77.9% (*P* < 0.001), respectively. On the other hand, when comparing the usual classification of one to three foci versus ≥4 foci, there was no statistically significant difference in CSS (*P* = 0.597) or DFS (*P* = 0.311).

Recently, it has been indicated that the presence or absence and degree of vascular invasion may have a prognostic impact, even in widely invasive FTC (34). In 2022, Ito *et al.* investigated the prognostic factors of an entire series of FTC in 523 patients (46). The 10-year distant recurrence-free survival rates of patients with minimally invasive FTC and widely invasive FTC without vascular invasion were 96.5% and 97.0%, respectively; the difference was not statistically significant (*P* = 0.127).

Yamazaki *et al.* investigated the prognostic factors for FTC with the incorporation of the histological subtype and degree of vascular invasion among 474 patients (37). The study included 140 patients with minimally invasive FTC, 260 with encapsulated angioinvasive FTC, and 74 with widely invasive FTC. Among the 474 patients, none died from FTC without vascular invasion during the follow-up period. Among the 428 patients with M0 FTC, the 10-year DMFS rates of patients with minimally invasive FTC and widely invasive FTC without vascular invasion were 99.1% and 100%, respectively, and the difference was not statistically significant (*P* = 0.515). Additionally, patients with WI-FTC and ≥2 vascular invasions had the worst 10-year DMFS rate (53.3%). Although the optimal cutoff number of vascular invasions is controversial, the presence and extent of vascular invasion are becoming increasingly important in predicting the prognosis of FTC.

### *TERT* promoter mutation

Recently, the presence of *TERT* promoter mutations has become more important for predicting prognosis and selecting additional treatments (47). Park *et al.* investigated CSS and DFS risk prediction using *TERT* promoter mutations in combination with histological subtypes among 77 patients with FTC (47). The study included 39 patients with minimally invasive FTC, 24 with encapsulated angioinvasive FTC, and 14 with widely invasive FTC. They produced the following three alternative groups: group 1 (minimally invasive FTC with wild-type TERT and mutant type TERT; encapsulated angioinvasive FTC with wild-type TERT), group 2 (widely invasive FTC with wild-type TERT), and group 3 (encapsulated angioinvasive FTC with mutant TERT; widely invasive FTC with mutant type TERT). The 15-year CSS rates for patients with minimally invasive FTC, encapsulated angioinvasive FTC, and widely invasive FTC were 95.5%, 78.3%, and 55.6%, respectively (*P* = 0.015). In contrast, among the alternative groups, the 15-year CSS rates for patients in groups 1, 2, and 3 were 94.6%, 66.7%, and 18.8%, respectively (*P* < 0.001). Therefore, this study indicated that *TERT* promoter mutations can be incorporated into the WHO system to better predict the prognosis of patients with FTC.

### Histological subtype

Widely invasive FTC is deemed to have a worse prognosis than minimally invasive or encapsulated angioinvasive FTC, the subset of prognostic factors associated with widely invasive FTC may be analyzed separately from minimally invasive or encapsulated angioinvasive FTC (28, 32, 48, 49).

A recent study by Yamazaki *et al.* investigated the prognostic factors in 107 patients with widely invasive FTC (34). The 10-year CSS rate in the 107 patients with widely invasive FTC was 94.3%. A multivariate analysis identified M1 (HR = 9.366, 95% CI: 1.082–81.03, *P* = 0.042) as an independent negative prognostic factor for CSS. Among 72 patients with M0 widely invasive FTC, the 10-year DMFS rate was 74.2%. A multivariate analysis showed that the number of VI C 2 (HR = 9.137, 95% CI: 1.165–71.67, *P* = 0.035) and resection margin status (HR = 5.853, 95% CI: 1.465–23.39, *P* = 0.012) were independent negative prognostic factors for DMFS.

In a study by Yamazaki *et al.* (37), the 10-year CSS rates in patients with minimally invasive FTC, encapsulated angioinvasive FTC, and widely invasive FTC were 100%, 97.5%, and 92.3% (*P* < 0.001), respectively. The 10-year DFS rates of patients with minimally invasive FTC, encapsulated angioinvasive FTC, and widely invasive FTC were 97.3%, 84.2%, and 69.9%, respectively (*P* < 0.001). Furthermore, patients with widely invasive FTC and vascular invasion were older and had more vascular invasion and higher thyroglobulin levels than patients with encapsulated angioinvasive FTC. Therefore, the present histological subtypes were appropriate. Some widely invasive FTCs, especially in patients with capsular invasion alone, may have a good prognosis (34, 37, 46). Furthermore, some encapsulated invasive angioinvasive FTCs, especially in patients with more vascular invasion, may have a poorer prognosis than some widely invasive FTCs (37, 46). Since widely invasive FTCs often have other negative prognostic factors, such as higher age, larger tumor size, a greater number of vascular invasions, and M1, there is no doubt that the histological subtype is an important prognostic factor in FTC. However, the prognosis of FTC can be stratified more accurately by combining the histological subtypes with other prognostic factors.

**Figure 1:**
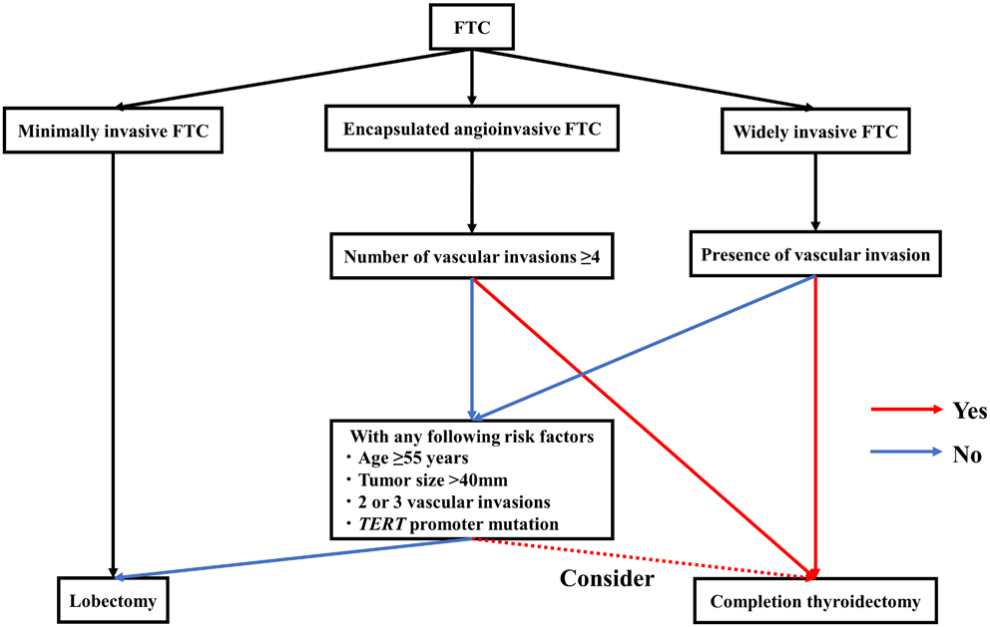
Proposed initial management flow of follicular thyroid carcinoma. FTC, follicular thyroid carcinoma.

The prognostic factors are summarized in Table 1. The proposed initial management flow for FTC is illustrated in Fig. 1.

**Table 1:** Summary of prognostic factors.

Study	Year	Histological subtype			Independent risk factors
		MI	EA	WI	
Shen *et al.* (49)	2024	118		35	Histological subtype, M1, tumor size
Yamazaki *et al.* (37)	2024	140	260	74	Age, histological subtype, number of vascular invasions, tumor size
Leong *et al.* (48)	2023	139	141	12	Age, histological subtype
Yamazaki *et al.* (34)	2023			107	M1, number of vascular invasions, resection margin status
Yamazaki *et al.* (31)	2022		264		Age, number of vascular invasions
Ito *et al.* (46)	2022	267	117	138	Age, non-oxyphilic, number of vascular invasions, vascular invasion, wide capsular invasion, sex
Yamazaki *et al.* (30)	2021	115	363		Age, tumor size

EA, encapsulated angioinvasive; MI, minimally invasive; WI, widely invasive.

## Treatment of advanced thyroid cancer

In the NCCN guidelines, nine drugs or drug combinations have been described for progressive and/or symptomatic FTC (NCCN guidelines version 1.2024 Thyroid Carcinoma. Available from: https://www.nccn.org/home (accessed 18 March 2024)). Anti-angiogenic drugs are multikinase inhibitors, including sorafenib, lenvatinib, and cabozantinib. Targeted agents include selective RET inhibitors (selpercatinib or pralsetinib) for FTCs harboring RET fusions or *RET* mutations, NTRK inhibitors (entrectinib and larotrectinib) for FTCs with NTRK fusions, and the BRAF/MEK inhibitor combination (dabrafenib/trametinib) for FTCs with *BRAF V600E* mutations. Furthermore, pembrolizumab is considered for FTCs with a tumor mutational burden. As the most common driver mutation of FTC is the *RAS* mutation, lenvatinib may be used most frequently among these drugs. Previous studies have revealed that the effects of lenvatinib treatment are influenced by various prognostic factors, including age, tumor volume, patient condition including performance status, tumor-related symptoms, neutrophil-to-lymphocyte ratio, histology, and metastatic site (50, 51, 52, 53, 54, 55, 56). It is important to consider these prognostic factors when deciding whether or not to introduce drugs.

## Conclusion

FTC remains a difficult tumor to diagnose preoperatively. Therefore, the development of useful preoperative examinations, including imaging findings, tumor markers, and genetic testing, is expected. Additionally, evaluating prognostic factors, particularly the histological subtype, vascular invasion, and distant metastasis, is essential for prognostic prediction and precise management of patients with FTC. Finally, given that *RAS* is the most common driver gene for FTC, it is also important to develop RAS inhibitors.

## Declaration of interest

The authors declare that there is no conflict of interest that could be perceived as prejudicing the impartiality of the study reported.

## Funding

This work did not receive any specific grant from any funding agency in the public, commercial, or not-for-profit sector.

## Acknowledgements

We thank Japan Medical Communication (https://www.japan-mc.co.jp/) for editing this manuscript.
